# Supplementary material for: Adaptation and psychometric properties of Psychological Skills Inventory for Sport (PSIS-R5) in Latvian athletes: Insights and implications for practice
Source: PLoS One. 2025 May 29;20(5):e0325225. doi: 10.1371/journal.pone.0325225 (PMC12122024; doi:10.1371/journal.pone.0325225)
Supplement: S1 File — (PDF) [file pone.0325225.s001.pdf]

S1 Appendix: Psychological Skills Inventory for Sports: Latvian Adapted Version (PSIS-S5-L)

This supplementary material includes the Latvian-adapted version of the Psychological Skills Inventory for Sports (PSIS-S5-L) presented in both Latvian and English for reference.

Psiholoģisko prasmju aptauja sportā (PSIS-S5-L)

Instrukcija: šie apgalvojumi aptver dažādus treniņu un sacensību aspektus. Lūdzu, vadoties pēc savas personiskās pieredzes, atzīmējiet tabulā attiecīgo atbildi tajā skalas rangā, kas norāda jūsu piekrišanu apgalvojumam (atbilde var variēt no pilnīgas piekrišanas līdz pilnīgai nepiekrišanai apgalvojumam). Neizlaidiet nevienu aptaujas apgalvojumu.

		Pilnībā nepiekrītu	Drīzāk nepiekrītu	Neitrāli	Gandrīz piekrītu	Pilnībā piekrītu
1.	Man ir ļoti liela motivācija gūt labus rezultātus savā sporta veidā	○	○	○	○	○
2.	Ar citiem komandas biedriem es satieku ļoti labi	○	○	○	○	○
3.	Man uzvara ir ļoti svarīga	○	○	○	○	○
4.	Pirms sacensībām es bieži vien domās veicu savu sacensībās plānoto darbību	○	○	○	○	○
5.	Pat nelieli sīkumi var iedragāt manu pašapziņu	○	○	○	○	○
6.	Neilgi pirms sacensību starta mani samērā bieži pārņem panika	○	○	○	○	○
7.	Ja es esmu pieļāvis kļūdu, tad man ir grūtības to aizmirst un koncentrēties uz savu tālāko darbību	○	○	○	○	○
8.	Man patīk trenēties kopā ar saviem komandas biedriem	○	○	○	○	○
9.	Es bieži šaubos par savām sportiskajām prasmēm	○	○	○	○	○
10.	Ja es sāku sacensību laikā pieļaut kļūdas, tad mana pašpārliecība krītas ļoti ātri	○	○	○	○	○
11.	Manuprāt, komandas gars ir ļoti svarīgs	○	○	○	○	○
12.	Kad es sev domās iztēlojos savas darbības, tad es "redzu" sevi tās izpildām gluži it kā es skatītos videoierakstu	○	○	○	○	○
13.	Kad gatavojos sacensību startam, es mēģinu iedomāties, kādas sajūtas būs manos muskuļos sacensību laikā	○	○	○	○	○
14.	Mana pašpārliecinātība ir ļoti mainīga	○	○	○	○	○
15.	Kad es pieļauju kļūdas sacensību darbībā, tad es kļūstu ļoti satraukts	○	○	○	○	○
16.	Šobrīd manā dzīvē vissvarīgākais ir gūt panākumus savā sporta veidā	○	○	○	○	○
17.	Mans sporta veids ir visa mana dzīve	○	○	○	○	○

Latvian adapted version of Psychological Skills Inventory for Sport (PSIS-S5-L)

Instructions: These statements cover various aspects of training and competition. Based on your personal experience, please indicate your response in the table corresponding to the scale ranking that reflects your agreement with the statement (responses may range from complete agreement to complete disagreement with the statement). Do not skip any statements in the inventory.

		Completely disagree	Somewhat disagree	Neutral	Almost agree	Completely agree
1.	I am very motivated to do well in my sport	○	○	○	○	○
2.	I get along very well with other members of a team	○	○	○	○	○
3.	Winning is very important to me	○	○	○	○	○
4.	I often "rehearse" my perfo1mance in my head just before I perform	○	○	○	○	○
5.	It doesn't take much to shake my self-confidence	○	○	○	○	○
6.	I am often panic struck during those last few moments before I begin my performance	○	○	○	○	○
7.	When I make a mistake, I have trouble forgetting it and concentrating on my ongoing performance	○	○	○	○	○
8.	I enjoy working with teammates	○	○	○	○	○
9.	I have frequent doubts about my athletic ability	○	○	○	○	○
10.	When I begin to perform poorly, my confidence drops very quickly	○	○	○	○	○
11.	I think team spirit is very important	○	○	○	○	○
12.	When I mentally practice I "see" myself performing (well) just like I was watching a videotape	○	○	○	○	○
13.	When I am preparing to perform I try to imagine what it would feel like in my muscles	○	○	○	○	○
14.	My self-confidence jumps all over the place	○	○	○	○	○
15.	When I make an error in my performance, I become very anxious	○	○	○	○	○
16.	Right now the most important thing in my life is to do well in my sport	○	○	○	○	○
17.	My sport is my whole life	○	○	○	○	○
