# Supplementary material for: Adaptation and psychometric properties of Psychological Skills Inventory for Sport (PSIS-R5) in Latvian athletes: Insights and implications for practice
Source: PLoS One. 2025 May 29;20(5):e0325225. doi: 10.1371/journal.pone.0325225 (PMC12122024; doi:10.1371/journal.pone.0325225)
Supplement: S2 File — (PDF) [file pone.0325225.s002.pdf]

S2 Appendix: List of Excluded Items from the Original PSIS-R5 Inventory

The table below summarizes the items excluded during the adaptation of the PSIS-R5-L, their original factor allocations, and the psychometric and theoretical reasons for their exclusion, ensuring the instrument's validity and relevance to contemporary sport psychology practices.

Item no.	Original Item Content	Original Factor	Reason for Exclusion
**Q2**	I often have trouble concentrating during my performance	Concentration	Low loading, failed to meet psychometric threshold for factor structure.
**Q3**	I often dream about competition	Mental preparation	Low loading, item content less relevant to contemporary definitions of mental rehearsal.
**Q4**	I am very self-confident about my athletic skills	Self-confidence	Low loading, redundant with stronger retained self-confidence items.
**Q5**	I get very frustrated when a teammate is performing poorly	Team emphasis	Redundancy with retained items. Frustration toward teammates indirectly covered by team cohesion items.
**Q6**	I am more tense before I perform than I am during performance	Anxiety control	Low loading, tension regulation is better addressed by retained factors.
**Q7**	I try not to think about my performance during the twenty-four hours before game	Mental preparation	Low loading, avoidance strategies are not emphasized in modern sport psychology.
**Q8**	I experience frequent "hot streaks" in which my performance is unusually good	Concentration	Low loading, outcome-focused rather than skill-focused item.
**Q9**	I sometimes lack the motivation to train	Motivation	Cross-loading, ambiguous factor structure undermined construct clarity.
**Q11**	I am seldom so tense that it interferes with my performance	Anxiety control	Low loading, insufficiently distinct from other anxiety control items.
**Q14**	In most competitions I go in confident that I will do well	Self-confidence	Low loading and redundancy, overlaps conceptually with stronger retained self-confidence items.
**Q15**	I tend to perform better when I feel more tense that less tense	Anxiety control	Low loading, inconsistent with modern views on optimal arousal and performance.
**Q16**	When I am actually performing, I am almost totally unaware of the audience	Concentration	Low loading, weaker psychometric relevance for concentration dimension.
**Q17**	When I am performing poorly I tend to lose my concentration	Concentration	Low loading and redundancy, concept captured more clearly in retained items.
**Q19**	I concentrate more on my own performance than on the performance of the team	Team emphasis	Low loading, ambiguity in item content relative to teamwork constructs.
**Q22**	I would like to be more motivated	Motivation	Low loading, insufficient discrimination among levels of athlete motivation.
**Q23**	A minor injury or a bad practice can really shake my self-confidence	Self-confidence	Low loading, similar conceptual content to retained confidence items.
**Q24**	I set goals for myself and usually achieve them	Motivation	Cross-loading, content overlaps with multiple factors, reducing scale purity.
**Q25**	I sometimes feel intense anxiety while I am actually performing	Anxiety control	Redundancy, intense anxiety during performance addressed by stronger items (Q20, Q38).
**Q26**	During my performance, my attention seems to slip back and forth between what I am doing and other things	Concentration	Low loading, concept better captured by retained concentration-related items.

**Q29**	I spend a lot of energy trying to stay calm before a meet	Anxiety control	Redundancy, calming down before performance is addressed more effectively through retained visualization items (Q13, Q33, Q35), reflecting modern active psychological preparation strategies.
**Q32**	I worry a lot about making mistakes in an important meet	Anxiety control	Content redundancy, concern over mistakes integrated within broader self-confidence constructs.
**Q34**	I can usually remain confident even through one of my poorer performances	Self-confidence	Low loading, overlaps thematically with other self-confidence items.
**Q37**	When my team loses, I feel badly - no matter how well I did as an individual	Team emphasis	Low loading, marginal psychometric contribution to team emphasis factor.
**Q40**	I am good at controlling my tension level	Anxiety control	Low loading, tension regulation better measured by retained self-confidence and anxiety-related items.
**Q41**	My anxiety level drops rapidly as soon as I begin my performance	Anxiety control	Low loading, insufficient conceptual clarity for modern anxiety control theory.
**Q43**	I have always worked well with my coaches	Team emphasis	Low loading, item focuses on coach-athlete relationship, not strictly team emphasis.
**Q44**	I have faith in myself	Self-confidence	Low loading, redundant with retained, better-loading confidence items.
**Q45**	When it comes down to the last hours before a game I often wish that I was better prepared	Mental preparation	Low loading, preparation-related constructs better covered by visualization items.
