# Supplementary material for: Adaptation and psychometric properties of Psychological Skills Inventory for Sport (PSIS-R5) in Latvian athletes: Insights and implications for practice
Source: PLoS One. 2025 May 29;20(5):e0325225. doi: 10.1371/journal.pone.0325225 (PMC12122024; doi:10.1371/journal.pone.0325225)
Supplement: Attachment — Submitted filename: Respond to the Reviewers.docx [file pone.0325225.s004.docx]

**May 1, 2025**

Dear Reviewers,

We sincerely thank you for your thoughtful and constructive feedback on our manuscript. We greatly appreciate the opportunity to revise and resubmit our work to PLOS ONE. Below, we respond point-by-point to each of your comments. We have also revised the manuscript accordingly, with all changes tracked (marked in blue in file: Revised Manuscript with Track Changes) in the marked-up version and a clean version submitted separately.

**Reviewer #1 comments and author responses**

**Comment 1:** *The original version of the instrument was developed over 15 years ago. Over time, instruments often undergo adaptations to maintain their validity and practical utility. I understand that the reduction of items aimed to align the instrument with more contemporary approaches to assessing psychological skills. However, I consider that there was a significant change from the original instrument to the version presented in this adaptation, with item reduction, exclusion, and renaming of factors. As a result, the cross-cultural adaptation seems more like the construction of a new instrument. I suggest making clearer which items were excluded and which changed factors, rather than presenting only the remaining items.*

**Response:** Thank you for this valuable observation. We fully agree. To improve clarity, we included a new table titled “List of Excluded Items from the Original PSIS-R5 Inventory” in Appendix (S2 Appendix. (DOCX)). This table presents each excluded item along with its original factor and theoretical justification for exclusion (e.g., low loading, redundancy, conceptual misalignment). We also added explanatory text in the Results section (lines 270-278) and Discussion (lines 590-594; 648-653) to support this table.

**Comment 2:** *The authors mention that previous versions had psychometric issues. I suggest comparing the reliability and validity indices of the current version with those of previous versions. This could clarify whether the exclusion of items compromised or maintained the instrument’s internal consistency and discriminant and convergent validity.*

**Response:** A comparison with the prior Latvian adaptation (Fernāte, 2008) has been added to the Results section (lines 359-368). We note that while the earlier version had Cronbach’s alpha coefficients ranging from 0.46 to 0.52 for several subscales, the PSIS-R5-L now shows α values from 0.50 to 0.87, with three out of four scales above 0.70. Fit indices (CFI = 0.968, RMSEA = 0.045) further support the improved model.

**Comment 3:** *The justification for the exclusion of items and factors should be explicitly discussed based not only on the statistical results presented (e.g., low factor loadings, items with low discrimination) but also on theoretical arguments, demonstrating that the eliminated items no longer reflect psychological skills considered fundamental in contemporary sports, if applicable. The authors should reinforce that this adaptation retains the strengths of previous versions while optimizing the instrument for current contexts. I recommend that the authors include a table or a comparative section explicitly showing how the psychometric indices and factors of the current version compare with previous versions. Additionally, I suggest detailing the theoretical and practical impact of these changes, using studies or references that support the need for the instrument’s update.*

**Response:** Thank you for this valuable suggestion. In response, we have addressed this in multiple places. In Appendix, each excluded item is explained statistically and theoretically. In the Results and Discussion (e.g. lines 535-540), we elaborate on why specific factors such as Anxiety Control and Concentration were not retained, referencing changes in conceptual understanding of psychological skills and practical irrelevance in some modern training settings. We cite Weinberg & Gould (2023) and Jekauc et al. (2023) to support this shift (see References 42 and 41, respectively).

**Comment 4:** *Although the article follows rigorous standards for cross-cultural and psychometric adaptation, the stated objective of validating the instrument exclusively for the Latvian context is a limitation, as Latvia is a small country with limited participation in elite sports. This could restrict the practical and theoretical impact of the study. I suggest that the authors more clearly present the sports landscape in Latvia and broaden the potential of this adaptation by reformulating the objective. This would allow the instrument to become a reference not only for sports practice but also for scientific research in international settings. Limiting the study to Latvia may hinder the generalization of the results. It would be interesting to discuss how the findings could be applied to other cultural and sports realities or how future studies could replicate this adaptation in countries with different sports contexts.*

**Response:** Thank you for pointing this out. We have now expanded the Introduction (lines 143-149) and Discussion to describe Latvia’s sports landscape (e.g., small elite athlete pool, limited access to sport psychologists, reliance on self-regulation). We also revised the objective (lines 133-135) and Conclusion (lines 648-653) to emphasize the instrument’s broader theoretical and practical utility for other small nations or underrepresented athletic populations. The text now encourages future cross-cultural replications.

**Comment 5:** *The adapted instrument has the potential to be useful not only in Latvia—especially since the article is published in English in this journal, which further emphasizes its potential for international use—considering the growing need for standardized assessments of psychological skills in sports. Expanding the scope could be further discussed as a next step in the research. Rather than limiting the objective to the Latvian context, the authors could discuss how the study contributes to advancing the theory of psychological skills in sports and to sports practice in general, regardless of national context.*

*Therefore, I recommend that the authors revise the objective to highlight the broader applicability of the instrument, emphasizing its theoretical and practical value in global sports contexts. This would not only strengthen the study’s relevance but also increase interest from the international scientific community.*

**Response:** Thank you for this insightful recommendation. We fully agree that the PSIS-R5-L adaptation holds potential beyond the Latvian context, particularly in light of the growing demand for culturally sensitive yet standardized tools in international sports psychology. In response to your comment, we revised the study aim section in the Introduction (lines 96-99 and 133-135) to clarify that, while the immediate adaptation was carried out for Latvia, the methodological approach and resulting instrument may also serve as a model for broader international adaptation efforts. Additionally, we expanded the Conclusion (lines 648-653) and Practical Implementation section (lines 670-673) to emphasize the relevance of the PSIS-R5-L for global use. These revisions underscore the theoretical and practical value of the tool across diverse sporting and cultural settings and highlight the potential for future cross-cultural validations. We hope this expanded framing addresses your suggestion and enhances the international relevance of our study.

**Reviewer #2 comments and author responses**

ABSTRACT:

**Comment 1:** *Focus on Practical Implications: The abstract could place more emphasis on the practical implications of the study, perhaps by mentioning how the adaptation of the PSIS-R5 could benefit coaches and psychologists in the Latvian sports context.*

**Response:** Thank you for this helpful suggestion. We revised the Abstract (lines 24-25 and 37-40) to highlight that the adapted PSIS-R5-L offers practical value to coaches and psychologists for evaluating and enhancing psychological skills in Latvian athletes.

**Comment 2:** *More Concise Summary: Although the abstract is well-written, it could be a bit more concise, focusing primarily on the key conclusions and practical implications.*

**Response:** We have refined the abstract to focus on the study's key objectives, core findings, and practical implications while maintaining necessary methodological detail. We believe these revisions improve clarity and focus for international readers (lines 37-40).

INTRODUCTION:

**Comment 3:** *The text could perhaps provide more details about the specific characteristics of the Latvian athlete population or how these characteristics might influence the development of psychological skills. Although the text mentions that the adaptation of the PSIS-R5 to Latvia is necessary, the section "there is a significant gap due to a lack of culturally relevant and modern instruments for accurately assessing psychological factors without the risk of outdated findings" [lines 130-131] does not deeply explain what the specific characteristics of the Latvian population are or the challenges these athletes face in relation to psychological assessment. It is suggested that more information about the target population could better contextualize the relevance of the study.*

**Response:** We have expanded the Introduction to provide a detailed description of the Latvian athlete population. We now explain the unique challenges faced by Latvian athletes, such as combining sport with academic or professional responsibilities, limited access to psychological support, financial constraints, and fewer international competition opportunities. These contextual factors emphasize the importance of culturally sensitive psychometric tools. We believe this addition strengthens the justification for adapting the PSIS-R5 inventory to the Latvian sports environment (lines 142-149).

METHOD:

**Comment 4:** *1. Face validity is a simpler and initial form of validation. While it is a useful step, it does not guarantee that the instrument is truly measuring the construct in a robust and accurate manner. Was any other form of validation conducted? Given the response, would it not be more prudent to consider this as an initial validation of the instrument?*

**Response:** Thank you for your observation. We agree that face validity alone is insufficient to confirm construct validity. In addition to the initial face validity check during translation, we conducted a comprehensive psychometric validation of the PSIS-R5-L, including Principal Component Analysis (PCA), confirmatory model fit indices (CFI = 0.968, RMSEA = 0.045, SRMR = 0.026), Cronbach’s alpha, Intraclass Correlation Coefficients (ICC), and newly added Composite Reliability (CR) and Average Variance Extracted (AVE) indicators. These analyses provide strong evidence of internal consistency, construct validity, and temporal stability. Based on these findings, we consider the PSIS-R5-L a psychometrically robust instrument rather than an initial validation. We have clarified this more explicitly in both the Methods and Results sections (lines 228-233 and 350-357).

**Comment 5:** *The sample of 444 athletes is sufficiently large, but it could be more detailed regarding the representation of different skill levels (elite, pre-elite, and amateur). Based on this, can the elite group be considered representative? 25 x 219 x 200? A more balanced sample in relation to skill level would be necessary to ensure the robustness and reliability of the conclusions.*

**Response:** We agree that the elite group (n=25) is relatively small compared to the pre-elite (n=219) and amateur (n=200) groups. This imbalance reflects the actual distribution of elite athletes in Latvia, where the population is small and the number of athletes meeting strict elite criteria (e.g., international competition, high training load, ≥5 years of experience) is limited. While the elite group size is modest, we ensured that it met the minimum sample size requirement for group comparisons using G*Power calculations. Moreover, we have now added a clarification in the text acknowledging this limitation and recommending that future studies increase elite representation to strengthen generalizability. Despite this, the significant differences identified suggest that the instrument is sensitive enough to detect psychological skill distinctions across performance levels. In the revised manuscript, we now explicitly address this point, noting that the elite sample size reflects the real distribution of athlete levels in Latvia, a country with a relatively small population and limited elite athlete pool (lines 607-616).

**Comment 6:** *Are the 40 individuals in the test-retest sample representative of the overall sample (elite, pre-elite, and amateur)?*

**Response:** Thank you for raising this point. The 40 athletes included in the test-retest reliability analysis were drawn proportionally from the larger sample to reflect representation across all three achievement levels (elite, pre-elite, and amateur)., Care was taken to ensure that the test-retest group included variability in sport type, gender, and experience. This approach was intended to preserve heterogeneity and strengthen the generalizability of the temporal reliability findings. We have added a clarifying sentence to the Methods section under Participants to reflect this (lines 159-160).

RESULTS:

**Comment 7**:*1Would it not be appropriate to give special attention to the visualization scale (Cronbach’s alpha of 0.5)? As it stands, could this limit the reliability of the visualization measure? The cited reference (22) does not claim that 0.50 is acceptable; rather, it states that improper use of these coefficients may lead to incorrect interpretations about the homogeneity and internal consistency of the tests. Therefore, the authors could reference a source that supports the 0.50 value. Reference 22 itself points out:*

*“The range of reliability measures are rated as follows: a) Less than 0.50, the reliability is low, b) Between 0.50 and 0.80, the reliability is moderate, and c) Greater than 0.80, the reliability is high” (Salvucci, Walter, Conley, Fink, & Saba, 1997: 115).*

**Response:** Thank you for this important observation. We agree that the initial wording regarding Cronbach’s alpha thresholds required clarification. Following your recommendation, we have revised the manuscript to more accurately reflect the interpretation provided by Salvucci et al. (1997). Specifically, we now state that coefficients between 0.50 and 0.80 are considered to indicate moderate reliability, rather than simply labelling values above 0.5 as acceptable. We have updated both the Methods section and the Reliability Analysis section accordingly to align with the referenced literature. We believe this change improves the precision and transparency of the manuscript (lines 228-233; 350-357).

**Comment 8:** *The sample of 444 athletes may not reflect the full diversity of the athletic population in terms of age, ethnic background, and number of sports represented. It is suggested that the authors detail how many athletes from each skill level are represented in each of the subgroups. For example: Were all elite athletes from the same sport? Age? Gender? Such information could directly influence the results obtained, such as self-esteem.*

**Response:** Thank you for your valuable observation. We fully agree that athlete demographics, such as sport type, age, and gender, could influence psychological skills. However, due to the relatively small number of elite athletes in Latvia, providing detailed subgroup characteristics (such as specific sports, exact age distribution, or gender breakdown) would risk compromising participant anonymity and confidentiality. To address your concern, we have clarified in the manuscript that elite athletes in the sample represented both team and individual sports and included both male and female athletes. Additionally, we have acknowledged this limitation in the Discussion section, noting that future studies with larger elite samples could provide a more detailed subgroup analysis while preserving confidentiality (lines 607-616).

DISCUSSION:

**Comment 9:** *The absence of Anxiety Control and Concentration scales, which are essential psychological skills at any level of sports performance, was mentioned but without a deeper exploration of the impact of this omission. It is suggested that this point be explored.*

**Response:** Thank you for this important observation. We have now expanded the discussion section to more explicitly address the impact of omitting the Anxiety Control and Concentration scales. Specifically, we highlight that while elements of these constructs are integrated into the Self-Confidence factor, the lack of dedicated subscales could limit the specificity of measurement. We also acknowledge this as a direction for future research, suggesting the potential supplementation of the PSIS-R5-L to more fully capture these essential psychological skills (line 535-540; 590-594)

**Comment 10:** *Although the study examines the applicability of the PSIS-R5-L, the discussion could delve deeper into the specific challenges faced by psychologists in real-world training and competition contexts, considering the different types of sports and competitive levels.*

**Response:** Thank you for this valuable suggestion. In response, we have expanded the Discussion section under "Psychologists' Perspectives on Elite Athletes' Psychological Skills" to include a more detailed reflection on the practical challenges psychologists encounter when working with athletes. This includes considerations of sport-specific psychological demands, access to support services, time constraints, and variability in athlete openness to assessment. We also emphasized how the PSIS-R5-L can be pragmatically used in settings with limited resources, such as Latvia, while acknowledging the need to tailor its interpretation across different sports and competition levels (607-616).

**Comment 11:** *The sample of elite athletes, which limits the depth of the analysis, could be further explored. The study acknowledges this limitation, but it could provide more information on how this might affect the results.*

**Response:** Thank you for highlighting the issue regarding the sample size of elite athletes. We agree that this is an important consideration. In the manuscript, we have acknowledged this limitation in the Limitations section. We explain that although the overall sample size is notably large for the Latvian athlete population, the relatively small number of elite athletes (n=25) may limit the depth of analysis specific to this group (line 624-632). We also note that this reflects the realities of the Latvian sports context, where the elite athlete population is limited. Additionally, we have emphasized the importance of future research focusing on expanding the elite athlete sample to further strengthen the reliability and generalizability of findings related to this subgroup. We believe that the current text adequately addresses this concern while maintaining the overall strength and contribution of the study.

CONCLUSION:

**Comment 12:** *It is recommended that the authors emphasize the need for further studies that address the limitations outlined in this study in order to refine the instrument even more. The study may not be generalizable, and therefore, it is crucial to point this out in the conclusion, as different sports may require adjustments in the assessment of psychological skills, depending on the type of sport.*

**Response:** Thank you for this helpful suggestion. We agree that acknowledging the limitations of generalizability and the need for further validation across different sports contexts is important. In response, we have revised the final paragraph of the Conclusion section to reflect this point more explicitly. While the adaptation was based on a large and diverse sample (n=444), we now clarify that subgroup analyses, especially for elite athletes, are limited by smaller numbers. Additionally, we emphasize that the psychological demands may vary across different sport types, and future research should aim to replicate and refine the instrument in varied athletic populations and disciplines to enhance its robustness and international applicability (line 648-653 and 670-673).

Once again, thank you for your thoughtful and constructive feedback, which helped us substantially improve the clarity and scientific rigor of our manuscript. We hope the revised version meets your expectations.

Sincerely,

Dr. K.V. (on behalf of all co-authors)
